# A semi-automated multiplex high-throughput assay for measuring IgG antibodies against *Plasmodium falciparum *erythrocyte membrane protein 1 (PfEMP1) domains in small volumes of plasma

**DOI:** 10.1186/1475-2875-7-108

**Published:** 2008-06-12

**Authors:** Gerald KK Cham, Jonathan Kurtis, John Lusingu, Thor G Theander, Anja TR Jensen, Louise Turner

**Affiliations:** 1 Centre for Medical Parasitology at Department of International Health, Immunology and Microbiology, University of Copenhagen and Department of Infectious Diseases, Copenhagen University Hospital, Copenhagen, Denmark; 2 Centre for International Health Research, Rhode Island Hospital, Brown University School of Medicine, Providence, USA; 3 National Institute for Medical Research, Tanga Centre, Tanga, Tanzania

## Abstract

**Background:**

The level of antibodies against PfEMP1 is routinely quantified by the conventional microtitre enzyme-linked immunosorbent assay (ELISA). However, ELISA only measures one analyte at a time and requires a relatively large plasma volume if the complete antibody profile of the sample is to be obtained. Furthermore, assay-to-assay variation and the problem of storage of antigen can influence ELISA results. The bead-based assay described here uses the BioPlex^100 ^(BioRad, Hercules, CA, USA) system which can quantify multiple antibodies simultaneously in a small plasma volume.

**Methods:**

A total of twenty nine PfEMP1 domains were PCR amplified from 3D7 genomic DNA, expressed in the *Baculovirus *system and purified by metal-affinity chromatography. The antibody reactivity level to the recombinant PfEMP1 proteins in human hyper-immune plasma was measured by ELISA. In parallel, these recombinant PfEMP1 proteins were covalently coupled onto beads each having its own unique detection signal and the human hyper-immune plasma reactivity was detected for each individual protein using a BioPlex^100 ^system. Protein-coupled beads were analysed at two time points seven months apart, before and after lyophilization and the results compared to determine the effect of storage and lyophilization respectively on the beads. Multiplexed protein-coupled beads from twenty eight unique bead populations were evaluated on the BioPlex^100 ^system against pooled human hyper-immune plasma before and after lyophilization.

**Results:**

The bead^-^based assay was sensitive, accurate and reproducible. Four recombinant PfEMP1 proteins C17, D5, D9 and D12, selected on the basis that they showed a spread of median fluorescent intensity (MFI) values from low to high when analysed by the bead-based assay were analysed by ELISA and the results from both analyses were highly correlated. The Spearman's rank correlation coefficients (Rho) were ≥ 0.86, (P < 0.0001) for all comparisons. Bead-based assays gave similar results regardless of whether they were performed on individual beads or on multiplexed beads; lyophilization had no impact on the assay performance. Spearman's rank correlation coefficients (Rho) were ≥ 0.97, (P < 0.0001) for all comparisons. Importantly, the reactivity of protein-coupled non-lyophilized beads decreased with long term storage at 4°C in the dark.

**Conclusion:**

Using this lyophilized multiplex assay, antibody reactivity levels to twenty eight different recombinant PfEMP1 proteins were simultaneously measured using a single microliter of plasma. Thus, the assay reported here provides a useful tool for rapid and efficient quantification of antibody reactivity against PfEMP1 variants in human plasma.

## Background

The hope of developing a vaccine against malaria is based on evidence that clinical immunity to the disease is developed through repeated exposures over several years to the pathogen [1]. Several studies suggest that protective immunity to malaria develop partly through the acquisition of a wide repertoire of specific antibodies directed against the polymorphic antigen target, *Plasmodium falciparum *erythrocyte membrane protein 1 (PfEMP1) [2,3].

To date, anti-PfEMP1 antibody levels in human plasma samples have been measured using enzyme-linked immunosorbent assay (ELISA). As *P. falciparum *malaria predominantly affects individuals of young age, studies of malaria immunity rely on plasma samples from infants and toddlers. This creates a limitation in using ELISA as obtainable plasma volumes from these target groups are relatively small. In addition ELISA is time consuming and labor intensive. Recent technological advances have resulted in the development of high-throughput multiplex methods which enable the simultaneous detection of antibodies to multiple analytes in human plasma samples.

Vignali [4] described the use of the Luminex^100 ^system, a bench-top flow cytometer equipped with two low power laser beams and capable of performing 100 discrete assays simultaneously in a single well. Each bead set is impregnated with a unique ratio of red-to-infrared dyes. When excited, each bead set emits its own unique detection signal that can be resolved by the instrument. Molecules covalently coupled to the beads, such as recombinant PfEMP1 proteins, can be detected by the use of a biotinylated secondary antibody with phycoerythrin-conjugated streptavidin used as a reporter.

Several studies have reported the use of multiplex assays to measure cytokine levels in samples [5], antibody levels to protein antigens [6] and antibodies to multiple malaria vaccine candidate antigens [7].

The assay reported here for evaluating the antibody profile of human plasma samples is based on a multiplex of twenty eight recombinant PfEMP1 protein coupled beads, each bead population with its own unique detection signal. The assay, requires one microliter of plasma sample for measuring antibodies to all twenty eight recombinant PfEMP1 proteins, is reproducible, gives results comparable to ELISA and is high-throughput. Importantly, the coupled beads remained stable after lyophilization and storage at -80°C.

## Materials and methods

### Reagents

1-ethyl-3-[3dimethylaminopropyl] carbodiimide hydrochloride (EDC) and *N*-hydroxysulfosuccinimide (Sulfo_NHS) were purchased from Pierce Biotechnology (Rockford, IL). 2-[*N*-morpholino] ethanesulfonic acid (MES), Tween-20, bovine serum albumin (BSA) sodium azide, biotinylated anti-human IgG, biotinylated anti-V5 antibody and phycoerythrin conjugated streptavidin were purchased from Sigma-Aldrich, USA.

### Plasma samples

The hyper-immune plasma pool was made up of plasma from ten individuals from a malaria endemic area of Liberia. Twenty samples from Danes who have never had malaria were used to make up the naïve pool. Sixty individual plasma samples, twenty each from people living in the three Tanzanian villages Mgome, Ubiri and Magamba with high, moderate and low malaria transmission [8], respectively were also analysed.

### Protein expression

Protein expression was as described previously [9,10]. Briefly, primer pairs designed to contain restriction enzyme sites (See additional file 1) were used to amplify Cysteine-rich inter-domain regions (CIDR) and Duffy binding-like (DBL) domains from 3D7 genomic DNA. The digested PCR products were cloned into the *Baculovirus *vector, pAcGP67-A (BD Bioscience), which was designed to contain the V5 epitope upstream of a histidine tag in the C-terminal end of the construct. The identity of the cloned fragments was verified by sequencing. Linearized Bakpak6 Baculovirus DNA (BD Biosciences Clontech) was co-transfected with pAcGP67-A into Sf9 insect cells for generation of recombinant virus particles and histidine-tagged proteins secreted into the supernatant of infected High-Five insect cell were purified on Co^2+ ^metal-chelate agarose columns. Eluted products were dialysed overnight in PBS. The yield, integrity and purity of the recombinant proteins were estimated by analysis on SDS gel, comparing to BSA standards, and by western blotting using the anti-V5 antibody. All of the proteins coupled to the Luminex beads were estimated to be at or above 80% purity. The sizes of the different recombinant proteins ranged from 45 to 60 kDa.

### Covalent coupling of recombinant PfEMP1 proteins to beads

Carboxylated Luminex beads were covalently coated with the different PfEMP1 protein domains through an interaction of their carboxyl groups and the amino groups on the proteins following the procedure suggested by the manufacturer. Beads (1.25 × 10^7 ^beads/ml) were brought to room temperature, vortexed for one minute and transferred to Eppendorf^® ^tubes. The supernatant was removed after centrifugation for one minute at 16,000 × g. 1 ml of distilled water was added to the beads, vortexed to re-suspend followed by centrifugation for one minute at 16,000 × g. The beads were sonicated in a water bath sonicator into suspension and centrifuged for one minute at 16,000 × g. The supernatant was removed by a pipette and 1 ml of activation buffer (0.1 M NaH_2_PO_4_, pH 6.2) added to the pellet and vortexed to re-suspend. In separate tubes Sulfo_NHS and EDC were reconstituted to 50 mg/ml and 125 μl of each added to the beads, vortexed and incubated at room temperature for twenty minutes with inversions in the dark. The beads were centrifuged for one minute at 16,000 × g, re-suspended in 1 ml of 50 mM MES pH 5.0, centrifuged for one minute at 16,000 × g and the supernatant removed. The MES wash was repeated. The beads were re-suspended in 500 μl of MES. In separate tubes, the different protein samples (100 μg of each) were mixed with MES to a final volume of 500 μl and each was added to a separate bead population and incubated at room temperature for two hours in the dark with inversions. The beads were centrifuged for one minute at 16,000 × g and the supernatant removed. The beads were washed twice in 1 ml of PBS/TBN (0.02% Tween-20, 0.1% BSA and 0.05% sodium azide in PBS pH 7.4. The beads were re-suspended in 1 ml of PBS/TBN and stored at 4°C in the dark. To determine if coupling was effective, aliquots of the different bead sets were prepared for analysis as described below and analysed on the BioPlex^100 ^system.

### Analysis of coupled beads on the BioPlex^100 ^system

The coated beads were diluted 1:333 in Assay Buffer E (ABE buffer: 0.1% BSA, 0.05% Tween-20, 0.05% sodium azide in PBS pH 7.4) and 50 μl aliquots of were dispensed into the wells of a 1.2 μm filter bottom 96-well microtiter plate (MSBVS 1210, Millipore, USA) pre-wetted with ABE buffer. The beads in 96-well plates were washed three times with ABE using a vacuum manifold (Millipore, USA). Frozen plasma samples were thawed at room temperature, mixed by vortexing, and spun at 16,000 × g for five minutes to remove particulates. Plasma samples were diluted 1:80 in ABE buffer and 50 μl aliquots of diluted sample was added to the beads and incubated in the dark on a shaking platform at 1100 rpm for thirty seconds followed by 300 rpm for thirty minutes. Excess antibody was removed using a vacuum manifold followed by three washes in ABE. 25 μl of biotinylated human IgG detection antibody diluted 1:500 in ABE was added to the beads, incubated in the dark with shaking at 1100 rpm for thirty seconds followed by 300 rpm for thirty minutes and washed three times in ABE. 50 μl of phycoerythrin-conjugated streptavidin diluted 1:500 in ABE was added to the beads and incubated in the dark with shaking at 1100 rpm for thirty seconds followed by 300 rpm for ten minutes. Excess phycoerythrin conjugated streptavidin was removed followed by three washes in ABE. The beads were then re-suspended in 125 μl of ABE and analysed on the BioPlex^100 ^system. The reader was set to read a minimum of 100 beads with identical unique detection signal and the results were expressed as median fluorescent intensity (MFI). In order to determine whether the human IgG detection antibody bound non-specifically to the coated beads, the beads were analysed against naïve plasma sample from Danes who have not been exposed to malaria.

### Multiplexing and lyophilization of beads

Equal volumes of the coated beads were pooled together and mixed by vortexing. This bead mix was divided in half and one half was stored at 4°C in the dark. Sucrose and Tween 20 were added to the other half to 3% and 0.05% respectively, mixed by vortexing and single-use aliquots were lyophilized (adVantage, Wizad™ 2.0, Virtis) in polypropylene vials, sealed under nitrogen gas and stored at -80°C. Immediately prior to use, lyophilized beads were reconstituted with distilled water and used for analysis as described above.

### Enzyme-linked immunosorbent assay (ELISA)

Antibody reactivity levels to the recombinant PfEMP1 proteins were measured in an ELISA system as described by Jensen *et al *[11]. The wells of Maxisorp microtitre plates (Nunc, Roskilde, Denmark) were coated by overnight incubation at 4°C with 100 μl of the recombinant PfEMP1 protein (5 μg/ml) diluted in 0.1 M glycine-HCl (pH 2.75). The plates were emptied and any residual binding sites were blocked by addition of 200 μl of blocking buffer (1% BSA, 0.5 M NaCl, 1% Triton X-100, in phosphate buffered saline (PBS) pH 7.2) per well followed by thirty minutes incubation at room temperature on a shaker. 100 μl of plasma sample diluted 1:80 in blocking buffer was added in duplicate wells and incubated for one hour at room temperature on a shaker. Following four washes in wash buffer (0.5 M NaCl, 1% Triton X-100 in PBS pH 7.4), the plates were incubated for thirty minutes at room temperature with 100 μl per well of peroxidase-conjugated goat anti-human IgG (Dako, Glostrup, Denmark) diluted 1:3000 in blocking buffer. The plates were washed four times in wash buffer and 100 μl of *o*-phenylenediamine substrate (Dako) activated with H_2_O_2 _was added to each well. The plates were incubated in the dark at room temperature before adding 100 μl of 2.5 M H_2_SO_4_. Optical densities were measured at 492 nm (OD_492_). All samples were analysed in duplicate. On each assay microtiter plate, a reference positive control plasma pool was included in addition to negative control wells without plasma (background levels). Results were calculated as arbitrary ELISA units (EU) to account for plate-to-plate variation as described by Jensen *et al *[12]:

$$\frac{{\text{OD}}_{\text{sample}}-{\text{OD}}_{\text{background}}}{{\text{OD}}_{\text{reference pool}}-{\text{OD}}_{\text{background}}}\times 100\%$$

These same samples were analysed by the bead-based assay and the results compared to those from ELISA.

## Results

### Protein-coupled beads are recognized by hyper-immune plasma

**Figure 1:**
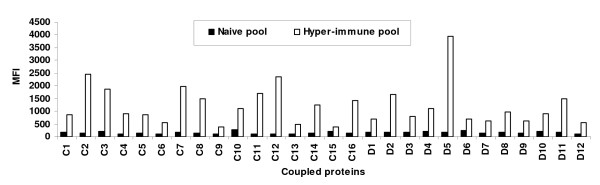
**Antibody reactivity to twenty eight recombinant PfEMP1 proteins in human plasma**. Twenty eight recombinant PfEMP1 proteins coupled onto beads with unique detection signals were analysed using a bead-based assay against a pooled hyper-immune plasma (white bars) and a pooled naïve plasma from Danish donors (black bars). The results were expressed as median fluorescent intensity (MFI).

Effective coupling of protein was determined by analysing the coupled bead populations against pooled hyper-immune plasma of ten Liberian individuals and pooled naïve plasma of twenty Danes on the BioPlex^100 ^system. Comparing the MFI obtained for each coupled domain analysed against both plasma pools showed a significantly higher MFI value for the pooled hyper-immune plasma in all cases (Figure 1). In addition the MFI values for the pooled naïve plasma were generally similar across the different bead population but varied in the case of the pooled hyper-immune plasma (Figure 1), suggesting heterogeneity in the levels of antibodies against the individual recombinant PfEMP1 proteins.

### Relationship between ELISA and bead-based assay

**Figure 2:**
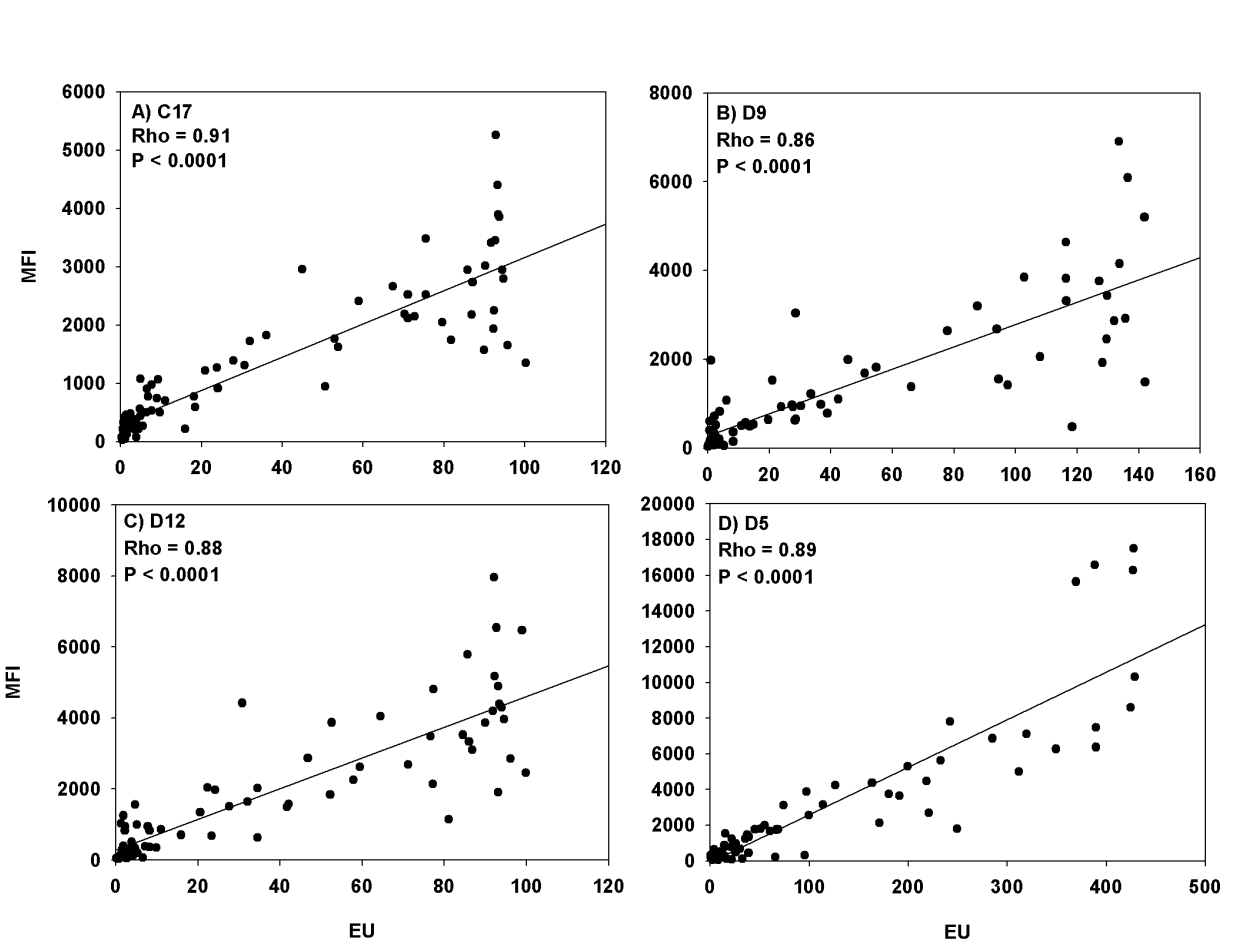
**Comparison of the bead-based assay and ELISA**. Four recombinant PfEMP1 proteins (C17, D5, D9 and D12) were analysed by ELISA and by the bead-based assay using twenty plasma samples from Danish donors and plasma samples from 60 individuals living in the three Tanzania villages Mgome (high malaria transmission), Ubiri (medium malaria transmission) and Magamba (low malaria transmission). The samples for both assay platforms were diluted 1:80 and results were expressed as median fluorescent intensity for the bead-based assay and arbitrary ELISA Units for ELISA. The Spearman's rank correlation coefficients (Rho) were ≥ 0.86, (P < 0.0001). Note: The graphs have different scales. MFI: median fluorescent intensity and EU: ELISA units.

Four recombinant PfEMP1 proteins C17, D5, D9 and D12 selected on the basis that they showed a spread of MFI values from low to high when analysed by the bead-based assay were chosen for this analysis. The results obtained from measuring antibody reactivity to these four proteins in eighty plasma samples (twenty individuals from each of three Tanzanian villages Mgome, Ubiri and Magamba and twenty Danes) by the bead-based assay were compared to those obtained by ELISA (Figure 2). For both analyses, the plasma samples were diluted 1:80. The Spearman's rank correlation coefficients (Rho) were ≥ 0.86, (P < 0.0001) for all comparisons. A detailed analysis to compare the sensitivity of ELISA and bead-based assay was not done. Using a cut off of mean plus two standard deviations of the value in the Danish control plasma, the percentage of responders amongst the African individuals were 70.0% vs. 66.6%, 48.3% vs. 65.0%, 63.3% vs. 71.7%, 86.7% vs. 48.3% in the bead-based assay vs. ELSA for proteins C17, D9, D12 and D5 respectively.

### Impact of multiplexing on protein-specific reactivity

**Figure 3:**
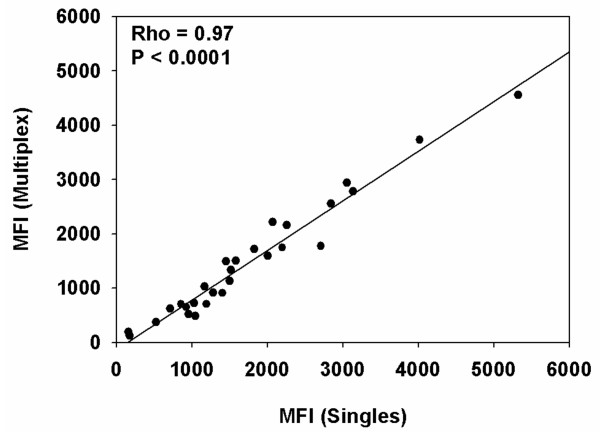
**Comparison of single bead and multiplex analysis**. Twenty eight recombinant PfEMP1 proteins coupled beads were analysed against the pooled hyper-immune plasma in single and multiplex formats. The results from both experiments were expressed as median fluorescent intensity (MFI). The Spearman's rank correlation coefficient (Rho) and P-value are shown.

To investigate the effect of multiplexing on antibody reactivity, the reactivity of pooled hyper-immune plasma with the coupled beads was measured in single and multiplex formats. The results from both were compared. The protein-specific MFI values obtained in each assay were close to identical, Spearman's rank correlation coefficient (Rho) = 0.97, (P < 0.0001)(Figure 3), indicating that in the multiplex format, the individual proteins on the beads do not compete for the available antibodies in the sample.

### Stability of protein-coated beads at 4°C

**Figure 4:**
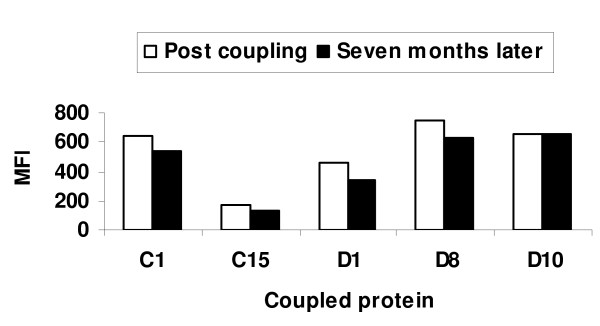
**The effect of long term storage at 4°C in the dark on coupled beads**. The median fluorescent intensity (MFI) values for five coupled bead populations were measured on the BioPlex^100 ^system against the pooled hyper-immune plasma just after coupling (white bars) and seven months later (black bars).

To determine the stability of the coupled beads after storage in the dark at 4°C, five protein-specific beads were analysed against the pooled hyper-immune plasma immediately after coupling (white bars) and after seven months of storage (black bars) and the MFI values from the two time points were compared (Figure 4). A slight decrease in MFI values after storage at 4°C was observed for all beads.

### Stability of protein-coated beads after lyophylization

**Figure 5:**
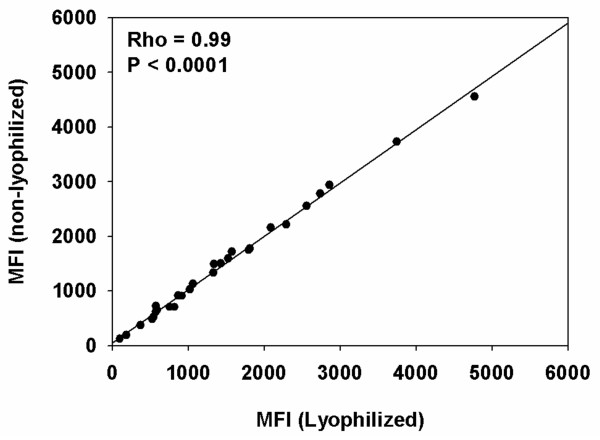
**Comparison of lyophilized and non lyophilized multiplexes**. The median fluorescent intensity (MFI) values for each coupled bead population in a multiplex of all twenty eight beads analysed against the pooled hyper-immune plasma before and after lyophilization of the multiplex. The Spearman's rank correlation coefficient (Rho) and P-value are shown.

To investigate the stability of the coupled beads after lyophilization the pooled hyper-immune plasma was screened with both lyophilized and non lyophilized coupled beads in a multiplex format and essentially identical results were obtained, Spearman's rank correlation coefficient (Rho) = 0.99, (P < 0.0001) (Figure 5). The inter-assay CV when measured in a single well immediately after lyophilization, three and six months later was within the limit reported below indicating stability of the lyophilized beads over time with storage at -80°C.

### Inter-assay and intra-assay variation for the bead-based assay

Inter-assay and intra-assay variation expressed as coefficient of variation (CV) were determined using the pooled hyper-immune plasma. The inter-assay CV was 13.4% when measured in a single well on ten consecutive plates. The intra-assay CV was 8.0% when measured in eight wells on a single plate.

## Discussion

One of the major challenges faced by researchers dealing with malaria in young children is the limited amount of plasma volume easily obtainable from infants and toddlers. Immunoassays such as ELISA require approximately 1 μl of sample for each analyte. In contrast, the multiplex assay has a major advantage of being able to theoretically analyse one hundred antigens simultaneously in 1 μl of sample. Fouda *et al *[7] reported a multiplex assay for measuring antibodies to nine *P. falciparum *malaria proteins. The multiplex assay described here measures antibody recognition to twenty eight recombinant *P. falciparum *erythrocyte membrane protein 1 (PfEMP1) proteins simultaneously in 1 μl of plasma sample. To demonstrate effective coupling, the coated beads were analysed individually against pooled hyper-immune plasma (Liberian individuals) and pooled naïve plasma (Danes) and were able to show a significantly higher recognition of the pooled hyper-immune plasma compared to the pooled naïve plasma for each individual coated bead (Figure 1). These results suggest that the recombinant PfEMP1 proteins were effectively coupled onto the beads.

Jacobsen [13] suggested guidelines for intra and inter-assay coefficients of variation of <10% and <20% respectively for bead-based assays. The bead-based assay reported here has intra- and inter-assay coefficients of variation of 8% and 13.4% respectively, well within the range suggested. Previous studies [7,14] suggest that the bead-based assay is more sensitive than ELISA in measuring antibody recognition in human plasma samples. For the protein D5, the sensitivity of the bead-based assay seemed to be markedly higher than in the corresponding ELISA assay, but for the three other proteins, the sensitivity of the ELISA assay seemed to be at level with or even a bit higher than the corresponding bead-based assay. The main difference between the results obtained in the two assays was seen in samples with high levels of antibodies, where ELISA readings seemed to have reached a sealed maximal value before the bead-based assay. In a few samples, there was a marked difference between the ELISA and bead-based assay readings (relatively low ELISA value/high bead-based assay values or vise versa). This could be due to the fact that different parts of the recombinant proteins are accessible by antibodies when the proteins are bound to a surface or a sphere. The major advantage of the bead-based assay over ELISA is its ability to measure several analytes simultaneously in a small volume of sample [15,16]. However, antibody competition and or blocking are to be expected if antigens that share antibody epitopes are detected together in a multiplex. To check for these all twenty eight PfEMP1 coupled beads were analysed in both single and multiplex formats and did not identify significant competition (Figure 3).

Stability of reagents is especially important if results are to be compared over an extended period. It has been shown that coupled beads remained stable for nine months with storage at 4°C [17]. There was a decreasing trend in the MFI values of coupled beads after storage at 4°C in the dark for seven months (Figure 4). However this problem can be resolved by lyophilizing the beads since MFI values for lyophilized beads do not differ significantly from those obtained using non-lyophilized beads (Figure 5). The lyophilization step is a novel approach to maintain the stability and extend the shelf life of coupled beads. This provides a major advantage of reducing batch-to-batch variation when for example analysing plasma samples that are collected over many years in longitudinal studies.

## Conclusion

The multiplex assay described here can measure simultaneously antibodies against twenty eight recombinant PfEMP1 proteins in human plasma. The assay also uses less sample volume, is faster and less labour intensive compared to ELISA. The assay shows a novel approach for maintaining the stability and extending the shelf life of coated beads thus reducing batch-to-batch assay variation. Thus, the assay described here provides a useful tool for studying the immunology of malaria especially in young children where the plasma volume available is usually limited.

## Competing interests

The authors declare that they have no competing interests.

## Authors' contributions

GKKC carried out the bead assay, did the data analysis and writing of the manuscript, JK participated in the study design, TGT, ATRJ, and LT participated in the study, data analysis and writing of the manuscript, JL collected the human plasma samples used. All authors read and approved the final manuscript.

## Supplementary Material

Additional file 1 Specific PfEMP1 domain primers used for PCR. Primers used to specifically amplify the PfEMP1 encoded fragments, the restriction enzyme sites are underlined and the extra bases precede the restriction sites. Click here for file
