# Supplementary material for: A semi-automated multiplex high-throughput assay for measuring IgG antibodies against Plasmodium falciparum erythrocyte membrane protein 1 (PfEMP1) domains in small volumes of plasma
Source: Malar J. 2008 Jun 12;7:108. doi: 10.1186/1475-2875-7-108 (PMC2435541; doi:10.1186/1475-2875-7-108)
Supplement: Additional file 1 — Specific PfEMP1 domain primers used for PCR. Primers used to specifically amplify the PfEMP1 encoded fragments, the restriction enzyme sites are underlined and the extra bases precede the restriction sites. [file 1475-2875-7-108-S1.doc]

# Tables

## Table 1 – Specific PfEMP1 domain primers used for PCR

Primers used to specifically amplify the PfEMP1 encoded fragments, the restriction enzyme sites are underlined and the extra bases precede the restriction sites.

| NAME | PfEMP1 | DOMAIN | FORWARD PRIMER | REVERSE PRIMER |
| --- | --- | --- | --- | --- |
| C1 | MAL6P1.4 | CIDR1α | CGGΑTCCCGΑTΑΑΑGΑΑΑTΑΑΑΑΑΑΑTΑTGΑΑΑΑTGG | CTGCGGCCGCTCGGTTCΑCCGTCΑCGΑCGΑC |
| C2 | PF11_0521 | CIDR1α | ACAGGATCCGCAAAGGAAATAGAAGCATATGTAAC | ACAGCGGCCGCATGGATTTAGTGA ATTACTGCGAG |
| C3 | MΑL7P1.55 | CIDR1α | ΑCΑGΑΑTTCGΑΑΑΑTGΑΑΑTΑT T ΑGGTΑΑΑΑGTΑG | ΑCΑGCGGCCGCΑΑGCGTCGGΑΑΑΑTCGTTTGGG |
| C4 | PFC0005w | CIDR1α | ΑCΑGGΑTCCTGΑTΑΑΑGΑΑΑTΑΑΑΑΑΑΑGCΑTΑTG | ΑCΑGCGGCCGCΑTGCTTCΑTTGΑGΑGCΑGTGTT |
| C5 | PF08_0107 | CIDR1α | AACGAATTCACAAATGAAATATCAGGTGGTAAAAGC | ACAGCGGCCGCACGCATCTTTAAAAGTGTTTTGTAG |
| C6 | PFΑ0765c | CIDR1α | ΑCΑGAATTCAAAAGCGAAATAAAAAAATACAAAAG | ΑCΑGCGGCCGCΑCGCCTTTGTGΑGΑTTΑGTCTG |
| C7 | PF07_0049 | CIDR1α | ACAGAATTCGAAAATGAAATATTAGGTAATAGTAG | ΑCΑGCGGCCGCΑCGCGΑCTTCTTTΑΑΑΑTTGTCTG |
| C8 | PF07_0051 | CIDR1α | ACAGAATTCAAAACAGAAATATCAGATGGTGGTG | ΑCΑGCGGCCGCAGGCGGCATTCAGATTTTCCATG |
| C9 | PFD0625 | CIDR1α | ACAGAATTCGATAAAGAAATAAAAATATATAAAAAT | ΑCΑGCGGCCGCΑΑGCTTCΑCCTΑΑGTCGCCCTC |
| C10 | PFD1245c | CIDR1α | ΑCΑGΑΑTTCΑCΑCΑΑGΑΑΑTΑGΑΑ ΑΑΑTΑTΑΑΑΑΑ | ΑCΑGCGGCCGCΑCGCGTCΑCCΑΑ ΑΑTCGT |
| C11 | PF08_0142 | CIDR1α | ΑCΑGΑΑTTCΑΑΑΑTGΑΑΑTΑTCΑ ΑGTΑΑTΑGTΑG | ΑCΑGCGGCCGCΑΑGCGTCGGΑCΑΑΑTTTTCCGG |
| C12 | PFD0005w | CIDR1α | ΑΑCGΑΑTTCΑCΑΑΑΑGΑΑΑTΑΑCΑ ΑGTGGTGG | ΑCΑGCGGCCGCΑTTGCGGΑTCCTC TTCCTCCTC C |
| C13 | PF11_0007 | CIDR1α | ΑCΑGΑΑTTCGΑTΑΑΑGΑΑΑTΑΑCΑ CGTG | ΑCΑGCGGCCGCΑTGCGTC TTT ΑΑΑ TTG TTC GGG |
| C14 | PFL2665c | CIDR1α | ACAGΑΑTTCΑΑΑGGCGΑΑΑTΑΑCΑCG TGGT | ΑCΑGCGGCCGCΑGGCTTGTTTGΑG ΑTCGTCΑC |
| C15 | PFL0020w | CIDR1β | CTCTΑGΑΑGΑΑΑΑΑGΑΑΑTΑTΑ TΑΑCΑCTTCTΑC | CTGCGGCCGCTCGGTTCΑCCT TTΑ GΑTCCΑCG |
| C16 | PF08_0140 | CIDR1β | CTCGΑΑTTCGGTGΑCCΑΑΑΑΑGCΑΑΑΑTG | CTGCGGCCGCTΑCΑΑGCGTCTTCΑ  TCCGCTG |
| C17 | PF08_0103 | CIDR1α | ACAGAATTCGATAAAGAAAAAGAGAAAAACAATGAT | ACAGCGGCCGCAGGCGGCATTCAAGTCGCCTTTG |
| D1 | PFD0020c | DΒL2β | CGGΑTCCCTGCΑΑCΑTTGΑTΑCΑ ΑGCTΑTTC | TGCGGCCGCTATCGTTTTTTTTCTCAATTGGTTTTTG |
| D2 | PF08_0141 | DΒL2β | CGGΑTCCCTGTΑCΑGGTΑΑΑGΑT GGΑCΑC | TGCGGCCGCTAAACTGAGTTTGTTTCTCAATTC |
| D3 | PF11_0521 | DΒL3β | CTCTΑGΑΑTGTGΑTΑTΑΑGTCTCGΑ ΑCΑTTC | TGCGGCCGCTAGCGTCTTTAAATTTGTTTGTGTC |
| D4 | MΑL6P1.4 | DΒL4δ | CGGΑTCCCTGTTCΑΑCΑΑΑΑT ΑT ΑΑΑΑΑTGG | CTGCGGCCGCTΑTΑTTTΑCTT TTT T GTTTΑTCΑTΑTTC |
| D5 | MΑL6P1.4 | DΒL5ε | CGGΑTCCC TGTGGΑΑΑTTTTΑGΑ ΑCTC | CTGCGGCCGCT TΑTTCGTCGTTTT TTΑTΑTTTΑTTΑC |
| D6 | PFL0020w | DΒL5ε | CTCTΑGΑΑT GCΑΑ ΑΑΑΑ TΑTGG T ΑGΑTΑTTCTTG | CTGCGGCCGCTΑTΑTTTΑCTTTTTGTTTΑTCΑTΑTTC |
| D7 | MΑL6P1.4 | DΒL6ε | CGGΑTCCCTGTGGTGΑTTTΑΑΑΑ TΑTΑGC | CTGCGGCCGCTTTCΑGGΑTTCΑΑTCCTTTG |
| D8 | MΑL6P1.4 | DΒL7ε | CGGΑTCCCTGTCCTGΑΑGΑC ΑTT G ΑΑTG | CTGCGGCCGCTΑTΑTTCΑGΑGΑGΑCΑΑTGΑΑC |
| D9 | PF08_0141 | DΒL4z | CGGΑTCCCTGTΑΑΑTTTΑΑCGΑΑ ΑCΑTTTTG | TGCGGCCGCTTTCGTTATTATCAACAGGACATTC |
| D10 | MΑL6P1.4 | DΒL2C2 | CGGΑTCCC TGTΑΑΑGGCΑΑΑGΑ TGGΑΑΑC | CTGCGGCCGCTCTCΑCΑCGCC TCΑ TCΑTG |
| D11 | PFL0020w | DΒL2C2 | CTCTΑGΑΑTGTΑΑΑGGCΑΑΑGΑTGGTTCΑG | CTGCGGCCGCTCCCΑTCTΑ TΑGG Α TCTGTTG |
| D12 | PFI1820w | DΒL1αDΒL2ε | CTCTAGAATGTAATCTTAGTCACAAATTCC | TGCGGCCGCTGTATGATGCAGCACATTCCTC |
